# Unlocking the human factor to increase effectiveness and sustainability of malaria vector control

**DOI:** 10.1186/s12936-021-03943-4

**Published:** 2021-10-16

**Authors:** April Monroe, Sarah Moore, Bolanle Olapeju, Alice Payne Merritt, Fredros Okumu

**Affiliations:** 1 Johns Hopkins Center for Communication Programs, Baltimore, MD USA; 2 University of Basel, Basel, Switzerland; 3 Swiss Tropical and Public Health Institute, Basel, Switzerland; 4 Ifakara Health Institute, Ifakara, Tanzania

## Abstract

**Background:**

Progress in the fight against malaria has stalled in recent years, highlighting the importance of new interventions and tailored approaches. A critical factor that must be considered across contexts and interventions is human behaviour.

**Main text:**

Factors such as acceptance of insecticide-treated nets (ITNs) and indoor residual spraying (IRS), ability and willingness to consistently use and appropriately care for ITNs and refraining from post-spray wall modifications can all impact the success of core vector control interventions. Understanding factors that can drive or inhibit these behaviours can contribute to improved social and behaviour change strategies and in turn, improved outcomes. Likewise, patterns of nighttime activities can reveal specific gaps in protection that cannot be filled by core interventions and inform development and deployment of complementary tools that meet people’s needs and preferences. There is an opportunity to increase use of approaches such as human-centred design to engage affected communities more actively in identifying and developing sustainable solutions that meet their needs and lifestyles. Integration of social and behavioural research with entomological and epidemiological evaluations will provide a more complete picture of malaria transmission dynamics and inform improved targeting of context-appropriate interventions. Finally, for gains to be maintained, interventions must be rooted within systems that support long-term success. This includes a movement toward more sustainable vector control solutions, increased decision-making and ownership of research, implementation, and strategy development at the country level, and inclusive approaches that ensure all men, women, boys, and girls are engaged as part of the solution.

**Conclusions:**

No matter how efficacious, a tool will remain ineffective if communities do not engage with it or use it regularly. Entering the next decade in the fight against malaria there is a critical opportunity to elevate the role of social and behaviour change to increase the impact and sustainability of malaria control and elimination efforts. This includes removing social and structural barriers to use of existing tools at all levels, human-centred and inclusive design and implementation of new tools, and movement toward long-term solutions led by affected communities.

## Background

Malaria, a parasitic infection transmitted by the female *Anopheles* mosquito, kills an estimated three people every 4 minutes [1]. Effective interventions, particularly those aimed at killing mosquitoes, or preventing biting, have significantly reduced malaria deaths [2]. Core vector control interventions, namely insecticide-treated nets (ITNs) and indoor residual spraying, are estimated to have contributed three-quarters of the reduction observed since 2000 [2]. Despite these gains, progress has plateaued in recent years and current approaches are unlikely to achieve 2030 global targets [3]. Persistent challenges, including below-target coverage and use of core interventions and widespread insecticide resistance, threaten fragile gains [1]. While new tools provide cause for optimism, achieving and sustaining malaria elimination will require greater understanding of a critical and often overlooked factor—human behaviour. This article highlights opportunities to optimize the effectiveness of core vector control tools, identify gaps in protection, ensure complementary interventions meet end-user needs, and enhance structural resilience against rebounds in malaria transmission (Box 1).

Box 1. Examples of opportunities to increase the impact and sustainability of vector control through improved understanding of human behaviour and resilience-building *Cross-cutting*

1. Implement evidence-based social and behaviour change (SBC) strategies to promote desired behaviours at individual, household, community, and societal levels.

*Optimize effectiveness of core vector control tools*

1. Ensure the benefits of ITNs resonate with families and that they are motivated to seek them out through available channels.
2. Consider improved ITN designs to increase usability, durability, and acceptance.
3. Use cues to action and nudges to promote consistent use and care practices.
4. Build trust through timely community engagement to increase acceptance of IRS activities.
5. Understand how product features impact acceptance of IRS and consider changes where possible to better fit needs and preferences of communities.

*Identify and characterize gaps in protection*

1. Integrate social and behavioural research with epidemiological and entomological data to identify and characterize context-specific gaps in protection.
2. Utilize human-centred approaches to understand day to day experiences of higher-risk groups and identify potential solutions.

*Develop appropriate complementary interventions*

1. Ensure new tools are developed with an understanding of people’s needs by engaging affected communities in design, evaluation, and implementation.
2. Integrate social and behavioural research when carrying out efficacy and effectiveness trials for new vector control tools.
3. Elevate the importance of social and behavioural considerations in policy evaluation and guidance for new tools.

*Build resilience to sustain gains*

1. Build capacity for, and ownership of, research, implementation, and monitoring of vector control activities among affected communities.
2. Increase domestic financing by endemic countries for malaria control.
3. Create structural resilience by improving homes and environments to sustainably suppress mosquitoes.
4. Encourage multi-sectoral approaches for disease prevention.
5. Integrate health education for disease control in school and community programmes.
6. Enhance participation in malaria programmes at all levels by identifying and addressing barriers to engagement for men, women, boys, and girls.

### Optimize core vector control tools

Today, insecticide-treated nets (ITNs) and indoor residual spraying (IRS) are the core vector control tools recommended by the World Health Organization (WHO) for wide-scale implementation against malaria [4]. Sustaining high levels of access, ensuring consistent use, and maximizing duration of effectiveness can increase impact, but are strongly dependent on the behaviours of individuals, households, and communities.

ITN distribution through mass and continuous distribution channels has led to a significant increase in access, however achieving and sustaining target-levels remains challenging [1]. Population access depends, in part, on people’s willingness to seek out ITNs at distribution points during mass campaigns, or through schools, health facilities, community leaders, or commercial sector channels. Effective social and behaviour change (SBC) approaches ensure that people know when and where to access ITNs and are able and willing to seek them out.

The protection provided by ITNs depends on consistent use, underscoring the importance of addressing barriers to use in settings, and among groups, with below-target levels. While reported use the previous night is generally high among those with access, ITN use is a complex behaviour that can vary by time of night, season, and over time [5]. A more holistic understanding of ITN use practices can inform effective SBC to increase ITN use and usability. This may also include development of innovative net designs and materials that expand the range of scenarios in which use is acceptable and feasible [6, 7].

Beyond access and use, people play an important role in determining how long ITNs remain effective and when and how they are discarded [8]. Different use patterns can contribute to ITN wear and tear, impacting longevity, and consequently cost effectiveness [8]. SBC can increase effective care practices, namely tying nets up when not in use and careful tucking and untucking, to extend longevity [9]. For both use and care which require daily action, habit formation can be encouraged through nudges and cues to action. Engaging youth through school programmes, and caretakers of young children through antenatal and immunization clinic visits, can likewise build life-long habits and an expanded net use culture.

Mosquitoes that rest or feed indoors can also be effectively controlled by IRS. Unlike ITNs, which currently rely primarily on pyrethroids, IRS includes a wider range of insecticide classes, allowing use in areas with pyrethroid resistance [10]. It is however logistically challenging and more expensive than ITNs and must, therefore, be deployed in targeted ways. To achieve necessary effectiveness, residents must welcome IRS sprayers into their homes and consent to the difficult task of removing household assets to allow insecticide treatment of walls. They must also be willing to avoid post-IRS modification of walls, such as covering with wall paper or decorations, that can impact the effectiveness of insecticides [11]. Effective community engagement, conducted in advance of spraying, can help achieve and maintain coverage. This includes timing spray activities to maximize the availability of residents at home. Changes to formulations, such as colourless IRS that reduces residual markings on walls, smart application in preferred mosquito resting locations to minimize the sprayed area, reduced frequency of application, and reduced odour, could increase acceptability and limit logistical constraints.

### Identify and characterize gaps in protection

While optimizing core vector control interventions is essential, these tools alone will not be sufficient to eliminate malaria. The effectiveness of IRS is limited when mosquitoes do not rest indoors and ITNs by biting that occurs outdoors or outside of sleeping hours. It is crucial to better understand activities that intersect with mosquito biting and determine where and when different sub-populations are exposed, what they are doing during these times, and specific approaches to limit risk [12, 13].

**Fig. 1:**
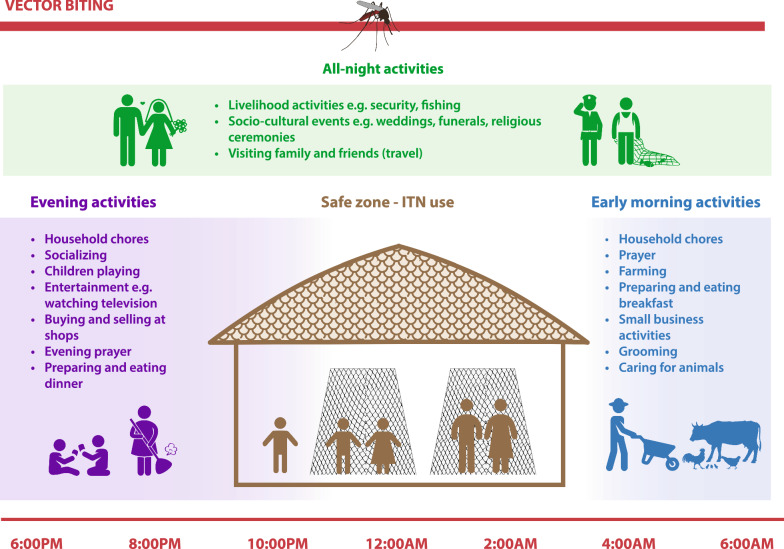
Illustration of gaps in protection that can occur even in the context of high coverage of core vector control tools. Adopted from Monroe et al. 2019 [14]

In many settings, gaps in protection arise during routine nighttime activities, such as household chores and socializing, that occur outdoors in the evening and activities like farming or fetching firewood or water that can occur early in the morning. Gaps can also occur during large socio-cultural events, such as weddings or funerals lasting all night where use of ITNs may not be feasible or socially acceptable [12] (Fig. 1). Similarly, nighttime occupations, travel, and migration can impact not only peoples’ exposure to malaria vectors but their ability to access and use core vector control interventions [12]. For example, seasonal workers travelling from higher transmission areas into lower transmission areas may be more likely to have an existing malaria infection upon arrival, may be less likely to have access to an ITN, and may stay or work in contexts where ITN use is more difficult [14]. Ensuring effective outreach and appropriate packages of services and interventions for higher-risk and mobile populations will be essential for reaching elimination. In these contexts, effective targeting of interventions is critical and more granular information on the epidemiological, ecological, and socio-cultural context is needed. Additional investigation of networks of higher-risk groups and travel patterns, and improved understanding of day-to-day experiences and priorities, can inform tailored approaches that better meet people’s needs.

### Develop appropriate complementary interventions

Complementary tools deployed alongside ITNs and IRS must be efficacious, readily available, and consistently used. A number of trials are planned or underway to help evaluate new vector control technologies, including Attractive Targeted Sugar Baits (ATSB), spatial repellents, systemic anti-parasitic drugs that kill biting mosquitoes, lethal house lures, and ITNs treated with new insecticide combinations [15]. These trials provide an opportunity to integrate social and behavioural research to better understand acceptability, barriers and motivators to use, changes in patterns of exposure to malaria vectors, and considerations for large-scale implementation. Examples of projects that are already integrating epidemiological, entomological, and social and behavioural data in the evaluation of new tools include the Advancing Evidence for the Global Implementation of Spatial Repellents (AEGIS) project [16], the Broad One Health Endectocide-based Malaria Intervention in Africa (BOHEMIA) project [17], and the New Nets project [18].

Active engagement of end-users has long been adopted by manufacturers of consumer products targeting nuisance biting and should be the norm for public health approaches, particularly for interventions with regular user touch points. Human-centred approaches that engage stakeholders at all levels in development and evaluation can ensure that new tools are closely aligned with people’s lifestyles, enabling a broader and more sustainable culture of use. This can include deploying empathetic research methods to fully understand the experiences and perspectives of end-users, working with end-users to define specific challenges to malaria prevention, co-creating and prioritizing solutions, and ensuring end-users test and provide ongoing feedback for new vector control approaches.

To ensure human behaviour is considered in development and evaluation of new technologies, it must be elevated within the policy review process. Currently, assessments address efficacy, quality, and safety, but not factors such as intended use patterns, end-user behaviours, or expected compliance levels [10]. Oftentimes user interaction data is collected by questionnaire or self-reporting, which may not provide a complete picture of user experiences. Further, participants may feel the need to please the researcher or appear in a certain light to their peers [19]. Complementary approaches based on in-depth conversations or observations, and integration of technology, such as motion sensors, to track and measure patterns of activity, can provide a clearer understanding of interactions with new tools [20]. Development of evaluation guidelines considering key human behavioural factors can help to encourage investment in tools that better meet the needs of families and communities. Once a new intervention is implemented, human behaviour indicators should also be considered in programme planning and reporting.

### Build resilience to sustain gains

ITNs, IRS and other commodities can provide short and medium-term gains, but long-term success will require sustainable solutions. Socioeconomic development has reduced the burden of malaria in parallel with malaria control efforts [1] providing an important opportunity to catalyse existing trends. Larval source management (LSM) and improved housing represent interventions that can be effective despite insecticide resistance and can be deployed by communities. LSM, which targets mosquito larvae through temporary or permanent changes to aquatic habitats, or use of chemical or biological larvicides, can reduce mosquitoes that bite indoors or outdoors [21]. Housing improvements, including screened windows and closed eaves, provide protection indoors and immediate nuisance biting reduction, without daily compliance. The proportion of Africans living in improved houses doubled between 2000 and 2015, with most improvements paid for by individual households [22]. Research on end-user design and material preferences, and the existing economy for housing materials and carpentry, can inform promotion, and where appropriate, subsidization, of mosquito-proof housing designs for disease control.

Vector control approaches must be nimble and adaptive to respond to changing local vector, disease, and demographic patterns. Such interventions should be locally owned and rely on agile implementation and monitoring systems. Capacity strengthening for data collection, management, analysis and use in-country, and social and behavioural research, can help to ensure availability and use of data at local levels. Increased domestic funding, with decision-making led by technical managers in-country, can minimize disruptions in access to commodities, foster resilience, and provide flexibility.

Sustainability will depend on full participation of affected communities. Gender norms, and intersecting sociodemographic factors, such as age and socio-economic status, play a relevant but often overlooked role in malaria-related behaviours and outcomes. Gender norms can influence a number of pathways to health, including differences in exposure, differences in health behaviours and access to care, differences in economic decision making, gender-biased health systems, and gender-biased research, institutions, and data collection [23]. Examples of how these pathways can influence malaria include differences in occupational risk associated with working at night or travelling for work, often for men, increased risk of exposure associated with nighttime or early morning household chores, often for women, differences in willingness to access or use prevention measures, differences in treatment received within the health system, and failure to collect and analyse data on differential experiences that can impact the success of interventions across different groups [5, 12, 24]. Programmes should strive to advance gender equality and social inclusion while promoting health and economic wellbeing for all. Examples include intersectional gender analysis to identify and address barriers to participation in malaria programmes at all levels, and inclusive, and when possible, transformative social and behaviour change approaches.

## Conclusion

Malaria outcomes and the success of interventions depend on access, acceptance, and consistent use of prevention measures. No matter how efficacious, a tool will remain ineffective if communities do not engage with it or use it regularly. Entering the next decade in the fight against malaria there is a critical opportunity to elevate the role of SBC to increase the impact and sustainability of malaria control and elimination efforts. This includes removing barriers to use of existing tools at all levels, human-centred and inclusive design and implementation of new tools, and a movement toward long-term solutions led by affected communities.

## Data Availability

Not applicable.

## Abbreviations

HCD: Human-centred design
IRS: Indoor residual spraying
ITN: Insecticide-treated net
SBC: Social and behaviour change
